# Medical Cases in Court

**Published:** 1887-06

**Authors:** Henry A. Riley

**Affiliations:** New York


### Medical Cases in Courts.

BY HENRY A. RILEY, ESQ., NEW YORK.

### MEDICAL CONTRACTS.

There was a recent case in the New Jersey Court of Chancery, on the interesting question whether a physician could make an agreement such as to prevent him from ever practicing medicine in a certain locality. It appeared that a physician, intending to be absent from the city of Newark a considerable time, employed another to attend to his practice, but fearing his patients might be lost to him when he resumed his practice, he secured the signature of the attending physician to this agreement: "In consideration of this contract made with him by the said * * * the said * * * hereby covenants and agrees not to engage in the practice of medicine or surgery in the city of Newark at any time hereafter." The employed physician did, after the termination of his engagement, begin practice for himself in Newark, and suit was brought to prevent him from doing so. The answer was that the agreement was an unreasonable one, and was in restraint of trade and void in law.

The court held that the claim was correct, and an injunction should not issue to prevent the physician from practicing for himself.

The main ground for the decision is that the agreement covered too long a period of time; it might be many years after the death of the physician who was supposed to be benefited. On this point the reasoning of the court will be interesting. The judge said: "The fault imputed to the covenant is that the restriction which it imposes is to endure for an unreasonable time --for a much longer period than will be necessary for the protection of the complainant. It interdicts the defendant, it will be observed, from practicing medicine or surgery in the city of Newark at any time hereafter. The restraint covers the whole period of defendant's life, and if an injunction is awarded enforcing the covenant according to its terms, the defendant can never at any time thereafter practice his profession in the city of Newark, though the complainant may the next year or even the next month after the injunction issues lose his life or his reason, or remove to another field of practice. Under such circumstances the injunction would give no protection to the complainant--he would need none; and the only purpose the injunction could serve would be to causelessly oppress the defendant."

